# Associations between persistent organic pollutants and type 1 diabetes in youth

**DOI:** 10.1016/j.envint.2022.107175

**Published:** 2022-03-16

**Authors:** Sophie E. Bresson, Scott Isom, Elizabeth T. Jensen, Sandra Huber, Youssef Oulhote, Joseph Rigdon, James Lovato, Angela D. Liese, Catherine Pihoker, Dana Dabelea, Shelley Ehrlich, Jérôme Ruzzin

**Affiliations:** a Department of Molecular Medicine, Institute of Basic Medical Sciences, Faculty of Medicine, University of Oslo, Oslo, Norway; b Wake Forest School of Medicine, Winston-Salem, NC, USA; c Department of Laboratory Medicine, University Hospital of North Norway, Tromsø, Norway; d Department of Biostatistics and Epidemiology, School of Public Health and Health Sciences, University of Massachusetts, Amherst, MA, USA; e Department of Epidemiology and Biostatistics, Arnold School of Public Health, SC, USA; f Department of Pediatrics, University of Washington, Seattle, WA, USA; g Lifecourse Epidemiology of Adiposity and Diabetes (LEAD) Center and Department of Epidemiology, Colorado School of Public Health, University of Colorado Anschutz Medical Campus, Aurora, CO, USA; h Division of Biostatistics and Epidemiology, Cincinnati Children’s Hospital Medical center, Cincinnati, OH, USA; i Department of Environmental Health, University of Cincinnati College of Medicine, Cincinnati, OH, USA

**Keywords:** Persistent Organic Pollutants, Type 1 Diabetes, Youths, Polychlorinated Biphenyls, Organochlorine Pesticides

## Abstract

**Background:**

Diabetes affects millions of people worldwide with a continued increase in incidence occurring within the pediatric population. The potential contribution of persistent organic pollutants (POPs) to diabetes in youth remains poorly known, especially regarding type 1 diabetes (T1D), generally the most prevalent form of diabetes in youth.

**Objectives:**

We investigated the associations between POPs and T1D in youth and studied the impacts of POPs on pancreatic β-cell function and viability *in vitro*.

**Methods:**

We used data and plasma samples from the SEARCH for Diabetes in Youth Case Control Study (SEARCH-CC). Participants were categorized as Controls, T1D with normal insulin sensitivity (T1D/IS), and T1D with insulin resistance (T1D/IR). We assessed plasma concentrations of polychlorinated biphenyls (PCBs) and organochlorine pesticides and estimated the odds of T1D through multivariable logistic regression. In addition, we performed *in vitro* experiments with the INS-1E pancreatic β-cells. Cells were treated with PCB-153 or p,p’-DDE at environmentally relevant doses. We measured insulin production and secretion and assessed the mRNA expression of key regulators involved in insulin synthesis (*Ins1*, *Ins2*, *Pdx1*, *Mafa*, *Pcsk1/3,* and *Pcsk2*), glucose sensing (*Slc2a2* and *Gck*), and insulin secretion (*Abcc8*, *Kcnj11*, *Cacna1d*, *Cacna1b*, *Stx1a*, *Snap25,* and *Sytl4*). Finally, we assessed the effects of PCB-153 and p,p’-DDE on β-cell viability.

**Results:**

Among 442 youths, 112 were controls, 182 were classified with T1D/IS and 148 with T1D/IR. The odds ratios (OR) of T1D/IS versus controls were statistically significant for p,p’-DDE (OR 2.0, 95% confidence interval (CI) 1.0, 3.8 and 2.4, 95% CI 1.2, 5.0 for 2nd and 3rd tertiles, respectively), *trans*-nonachlor (OR 2.5, 95% CI 1.3, 5.0 and OR 2.3, 95% CI 1.1, 5.1 for 2nd and 3rd tertiles, respectively), and PCB-153 (OR 2.3, 95% CI 1.1, 4.6 for 3rd tertile). However, these associations were not observed in participants with T1D/IR. At an experimental level, treatment with p,p’-DDE or PCB-153, at concentrations ranging from 1 × 10^−15^ M to 5 × 10^−6^ M, impaired the ability of pancreatic β-cells to produce and secrete insulin in response to glucose. These failures were paralleled by impaired *Ins1* and *Ins2* mRNA expression. In addition, among different targeted genes, PCB-153 significantly reduced *Slc2a2* and *Gck* mRNA expression whereas p,p’-DDE mainly affected *Abcc8* and *Kcnj11*. While treatment with PCB-153 or p,p’-DDE for 2 days did not affect β-cell viability, longer treatment progressively killed the β-cells.

**Conclusion:**

These results support a potential role of POPs in T1D etiology and demonstrate a high sensitivity of pancreatic β-cells to POPs.

## 1. Introduction

Recent estimates indicate that the US prevalence of diabetes continues to significantly increase among children and adolescents (Lawrence et al. 2021). Diabetes is a chronic disease characterized by an elevation of blood glucose levels as a result of insufficient insulin for the body’s needs. The resultant hyperglycemia is known to significantly increase the risk of heart disease, stroke, and microvascular complications (*e.g.* blindness, renal failure, and neuropathy) (Harding et al. 2019). In type 1 diabetes (T1D), hyperglycemia results from a loss of insulin production caused by pancreatic β-cell dysfunction and/or destruction as a result of an autoimmune process (Srikanta et al. 1983; Krogvold et al. 2015). In type 2 diabetes (T2D), hyperglycemia results from insulin resistance and reduced or insufficient compensatory insulin production (Muoio and Newgard 2008). Whereas lifestyle factors like diet and physical inactivity are strong regulators of insulin resistance (Kolb and Martin 2017), the environmental factors provoking the β-cell dysfunction and/or destruction remain poorly understood (Roep et al. 2021).

Persistent organic pollutants (POPs) are human-made chemicals that have been widely used to support industrial activities (Ruzzin 2012). Because of their high resistance to biodegradation and long half-life, POPs are very ubiquitous and are often referred to as the “*forever chemicals*”. Some examples of well-known POPs include organochlorine pesticides, which have been extensively used in agriculture, and polychlorinated biphenyls (PCBs), which have been massively used in hydraulic equipment, paints, plastics, and rubber products. Although most POPs have been banned for decades, some countries still produce and use these chemicals (Lopez-Carrillo et al. 1996; Jorgenson 2001; Sarker et al. 2021), and climate changes are likely to induce an uncontrolled release of POPs from ice and soil deposits during the coming years (Teran et al. 2012). Today, organochlorine pesticides and PCBs are omnipresent in our food products (Schecter et al. 2010; Sirot et al. 2012), primarily fish, meat, and dairy food products, and humans are consequently chronically exposed to these chemicals throughout their lifespan.

In humans, exposure to POPs, especially organochlorine pesticides and PCBs, has recently been associated with a higher risk of T2D (Lee et al. 2014). A link between POPs and T2D is further supported by preclinical evidence; for instance, dietary POPs derived from fatty fish impair insulin sensitivity (measured by hyperinsulinemic-euglycemic clamp) in rats by reducing insulin-stimulated glucose uptake in skeletal muscle and adipose tissue, and insulin-mediated suppression of glucose production in the liver (Ruzzin et al. 2010). Mechanistically, POPs can alter the insulin-stimulated glucose transporter GLUT4 (Olsen et al. 1994), impair insulin signaling pathways (Lyche et al. 2011; Singh et al. 2019), disrupt mitochondrial function (Tremblay-Laganiere et al. 2019; Lee et al. 2021), and promote chronic-low grade inflammation (Ruzzin et al. 2010), thereby contributing to insulin resistance. While the link between POPs and T2D is now well-established, the impacts of these environmental pollutants on T1D are less defined (Howard 2019) and the mechanisms underlying a potential effect of POPs on pancreatic β-cells remain largely unknown (Hectors et al. 2011). To address these knowledge gaps, we tested the hypothesis that POPs are associated with T1D and directly cause pancreatic β-cell dysfunction and/or destruction.

In the present study, we used stored samples from 442 participants in the SEARCH Case-Control (SEARCH-CC) study and measured the circulating concentrations of organochlorine pesticides and PCBs in participants aged 10–22 years diagnosed with T1D. Since youths with T1D were previously found to fall into two categories, T1D with normal insulin sensitivity (T1D/IS) and T1D with insulin resistance (T1D/IR) (Dabelea et al. 2011), we investigated whether organochlorine pesticides and PCBs were positively associated with T1D/IS and T1D/IR. In addition, we performed *in vitro* β-cell studies to further determine whether POPs directly affect insulin production and secretion, regulate gene expression, and affect the viability of β-cells.

## 2. Materials and Methods

### 2.1. Human study

We used data and samples collected in 2003–2006 from the SEARCH-CC study, an ancillary study to the multicenter study of diabetes in U.S. youth (Dabelea et al. 2011; Hamman et al. 2014). For POP analyses, we used serum samples that were collected following fasting and where triglyceride values were obtained. SEARCH-CC participants who were residents of Colorado or South Carolina, aged 10–22 years, were recruited from primary care offices, as described previously (Guy et al. 2009; Snell-Bergeon et al. 2010). Among SEARCH-CC participants, youths were classified as controls (no diabetes), recently-diagnosed T1D/IS, or recently-diagnosed T1D/IR (Dabelea et al. 2011). The nondiabetic status of controls was confirmed by normal fasting glucose values, normal insulin sensitivity, and absence of T1D autoantibodies. T1D was determined based on clinical diagnosis and medical record review and confirmed based on the positivity of one of three specific T1D autoantibodies measured; glutamic acid decarboxylase antibodies (GADA), insulinoma-associated 2 antibodies (IA-2A), and zinc transporter 8 antibodies (ZnT8) (Crume et al. 2020). For insulin sensitivity assessment, a clinical algorithm validated against hyperinsulinemic-euglycemic clamps was used with a cut-point of 8.15 for determining the state of insulin resistance (<8.15: insulin resistance, ≥ 8.15: insulin sensitive) (Dabelea et al. 2011).

### 2.2. POP analysis

Plasma samples from SEARCH-CC participants were shipped in frozen condition to the Laboratory for Analysis of Environmental Pollutants at the Department of Laboratory Medicine, University Hospital of North Norway, Norway, and stored at ≤ −20 °C prior to analysis for organochlorine pesticides and PCBs (Table S1). Fully validated methods were applied for sample preparation and instrumental analyses as described previously (Huber et al. 2020). A Tecan Freedom Evo 200 liquid handler (Männedorf, Switzerland) equipped with a solid-phase extraction station, a robotic manipulator arm and a shaker was used for sample preparation. Before extraction and clean-up by automated solid-phase micro-extraction, a 150 μL plasma sample was diluted and spiked with an internal mass-labelled standard solution. Instrumental analyses were performed by gas chromatography with atmospheric pressure ionisation coupled to tandem mass spectrometers (Waters, Milford, MA, USA). The atmospheric pressure ionization was conducted in positive mode under charge transfer conditions. For detection on the mass spectrometers, multiple reaction monitoring (MRM) mode with two specific transitions for the individual analytes was applied.

Masslynx and Targetlynx software (Version 4.1, Waters, Milford, MA, USA) was used for quantification by application of the isotope dilution method. For quality assurance, four blank samples, four SRM 1957 and SRM 1958 samples (both NIST, Gaithersburg, MD, USA), and three bovine serum samples (Sigma Aldrich, Steinheim, Germany) were analyzed within each batch of 96 samples to control for background and carry-over effects. The quality controls were within acceptable limits and similar to our previous data (Huber et al. 2020) with coefficients of variation (CVs) ranging from 4 to 28% for the present study. The laboratory participates successfully in the international quality control programme Arctic Monitoring and Assessment (AMAP) Ring Test for Persistent Organic Pollutants in Human Serum, organized by the Laboratoire de toxicologie, Institut National de Santé Publique du Quebec, Canada, with a satisfying performance reaching z-scores within the acceptable range of −2 > z-score < 2.

### 2.3. In vitro studies

Rat insulinoma INS-1E cells (Merglen et al. 2004) were cultured in a humidified atmosphere with a 95% air / 5% CO_2_ mixture and maintained at 37 °C as described previously (Ghiasi et al. 2019). Briefly, cells were grown in 11 mM glucose in GlutaMAX Roswell Park Memorial Institute (RPMI)-1640 medium (Life-Technologies, United Kingdom) supplemented with 10% fetal bovine serum, 100 IU/mL penicillin and 100 ug/mL streptomycin, 0.05 μM β-mercaptoethanol, 1 mM sodium pyruvate, and 10 mM hydroxyethyl piperazineethanesulfonic acid. At 90–95% confluence, cells (150,000 cells seeded in 24-well plates) were cultured with 11 mM glucose in RPMI-1640 medium for 45 h and treated with dimethyl sulfoxide (DMSO), p,p’-DDE (MW: 318.03, Sigma-Aldrich, Switzerland) or PCB-153 (MW: 360.88, Sigma-Aldrich, Switzerland) at environmental concentrations; 1 × 10^−15^ M (1 fM), 1 × 10^−12^ M (1 pM), 1 × 10^−9^ M (1 nM), 1 × 10^−6^ M (1 μM), and 5 × 10^−6^ M (5 μM). In all treatments, the maximal concentration of DMSO did not exceed 0.025%, a dose without any effect on cell viability (data not shown). In addition, TNFα (Novus Biologicals, USA) was used as a positive control (Kim et al. 2008). After 45 h, treated cells (DMSO, POPs or TNFα) were grown in RPMI-1640 medium containing 2 mM glucose for 1 h. Then, treated cells (DMSO, POPs or TNFα) were cultured with 20 mM glucose in RPMI-1640 medium for 2 h to stimulate insulin production. Thereafter, INS-1E cells were collected for assessment of intracellular insulin content and mRNA expression whereas culture media was used to measure glucose-stimulated insulin secretion. In total, cells were exposed to DMSO, PCB-153, p,p’-DDE, or TNFα for 48 h. In another set of experiments with similar treatments, INS-1E cells were plated in a 96-well plate (50,000 cells per well) and cultured with 11 mM glucose in GlutaMAX RPMI-1640 medium for 8 days to assess cell viability.

### 2.4. Insulin analyses

Secreted and intracellular insulin was measured using a Rat insulin ELISA (#10-1250-01, Mercordia, Sweden) from culture media and INS-1E cells, respectively. All results were normalized to protein content measured by Pierce BCA Protein Assay Kit (Thermo Scientific, USA). Samples were analyzed in duplicates for both ELISA and BCA analyses, and the average of these technical duplicates was used.

### 2.5. Gene expression analyses

Total RNA was prepared from INS-1E cells using the Invitrogen total RNA isolation kit (Life-technologies, USA). Complementary DNA generated with a high-capacity cDNA Reverse transcription kit (Thermo Scientific, Lithuania) was analyzed by quantitative real-time qPCR using the BioRad iQ^™^SYBR^®^ Green supermix (CFX Maestro Version 5.0.021.0616, BioRad, USA). Efficiency corrected Cq values were normalized to *Tbp* expression and expressed as fold change relative to DMSO-treated cells. Oligonucleotide primers are listed in Table S2.

### 2.6. Cell viability analyses

Cell viability was determined using the AlamarBlue^™^ HS cell viability reagent (Thermo Scientific, USA) according to the manufacturer’s instructions.

### 2.7. Statistical analyses

#### Human studies.

Characteristics of participants are described using mean and standard deviation, median and interquartile range (IQR), or counts and percentages depending on the distributions of the characteristic. For comparisons between the Control, T1D/IS, and T1D/IR groups, t-tests, Kruskal-Wallis tests, or exact tests based on the distributions were conducted as indicated in Tables.

In our investigations, POPs were not included in our analyses when more than 80% of their detection values were below the limit of detection (LOD). When distributions of the POPs were skewed a log transformation was applied to normalize the data prior to imputation. Multiple imputation of values below LOD was done using IVEWare for SAS (University of Michigan) with organochlorine pesticide compounds imputed separately from PCB compounds. Imputed values were constrained to be between zero and the LOD. Ten analytic datasets were created for each compound type for use in modeling. Included in the imputation process were the POPs and participant characteristics (diabetes type, age, gender, race and ethnicity, SEARCH site, parental education, income, insurance, total cholesterol, low-density lipoprotein (LDL) cholesterol, high-density lipoprotein (HDL) cholesterol, triglycerides, body-mass index (BMI) z-score, insulin sensitivity, total lipids, pubic Tanner stage). After imputation was completed, lipid-adjusted POP concentrations (POP/total lipids; total lipids = 2.27 × total cholesterol + triglycerides + 62.3) were divided into tertiles. We also performed analyses with non-lipid-adjusted POP concentrations. Multivariable logistic regression was used to compare differences in the tertiles between T1D/IS, T1D/IR, and the control group after adjusting for possible confounders (i.e. age at sampling, health insurance, parental education, race/ethnicity, sex, pubic Tanner stage, total lipids, and site), as informed by directed acyclic graphs (Fig S1). The odds ratios (OR; 95% confidence interval (CI)) use the control group and the lowest tertile of the POP concentration as reference groups. Additional models with the same covariates and the tertiles being treated as ordinal variables were run to test for a trend across the tertiles. Results of the models for the POPs with detection rates greater than 70% are shown as the primary results. Results of the models for the POPs with 20 to 70% detection rates can be found in supplementary Table S4.

#### In vitro studies.

Differences between groups were evaluated with a one-way ANOVA corrected for multiple comparisons and followed by post-hoc unpaired student t-tests. For viability cell experiments, differences were assessed using a two-way ANOVA, followed by unpaired *t*-test corrected for multiple comparisons. Data are presented as means with the standard error of the mean (SEM). P-values < 0.05 were considered to be statistically significant. All analyses and figures were made with Graph Pad Prism (version 8.3.0 for Windows, GraphPad Software, California, USA).

## 3. Results

### 3.1. Human study

The characteristics of participants are shown in Table 1. Among 442 youths, 112 youths had no diabetes (controls) whereas 330 youths had T1D; 182 and 148 youths with T1D had normal insulin sensitivity (T1D/IS) or insulin resistance (T1D/IR), respectively (Table 1). The mean age was 13.5 and 16.1 years for T1D/IS and T1D/IR, respectively (Table 1). As expected, youths with T1D/IS had a significantly lower BMI z-score, waist circumference, circulating lipids, and HbA1c, as compared with T1D/IR participants (Table 1). The duration of T1D was also longer in T1D/IR participants than in youths with T1D/IS (Table 1).

Next, we analyzed the plasma concentrations of different organochlorine pesticides and PCBs in the participants. Among organochlorine pesticides, p,p’-DDE, and hexachlorobenzene were detected in almost every participant regardless of case or control status, followed by *trans*-nonachlor (78% detection rate) (Table 2 and S3). Among PCBs, PCB-28 and PCB-153 were the most prevalent compounds with a detection rate close to 80% (Table 2 and S3).

After adjustment for confounders (age at visit when the sample was collected, health insurance, parental education, race, ethnicity, sex, pubic Tanner stage, and study site), there was a significant association between T1D/IS and several POPs including p,p’-DDE, *trans*-nonachlor and PCB-153 (Table 2). The odds of T1D/IS were significantly increased among youths in the 2nd (OR 2.0, 95% CI 1.0, 3.8) and 3rd tertiles (OR 2.4, 95% CI 1.2, 5.0) of serum p,p-DDE levels. Similarly, both the 2nd and 3rd tertiles of serum *trans*-nonachlor levels were associated with a significant elevation in the odds (OR 2.5, 95% CI 1.3, 5.0 and OR 2.3, 95% CI 1.1, 5.1 respectively). For serum PCB153 levels, the odds of T1D/IS were significantly increased among youths in the 3rd tertiles (OR 2.3, 95% CI 1.1, 4.6). Among POPs with detection rates between 20 and 70%, only the 3rd tertile of serum p,p’-DDT levels showed a significant increase of the odds for T1D/IS (OR 2.1, 95% CI 1.0, 4.3) (Table S4). Of note, these positive associations were not observed for youths with T1D/IR, and only heptachlor was positively associated with T1D/IR (OR 2.1, 95% CI 1.0, 4.7 for 2nd tertile) (Table 2 and Table S4). Analyses with non-lipid adjustments for POPs provided similar results (Table S5).

### 3.2. POPs and β-cell function in vitro

**Fig. 1.**
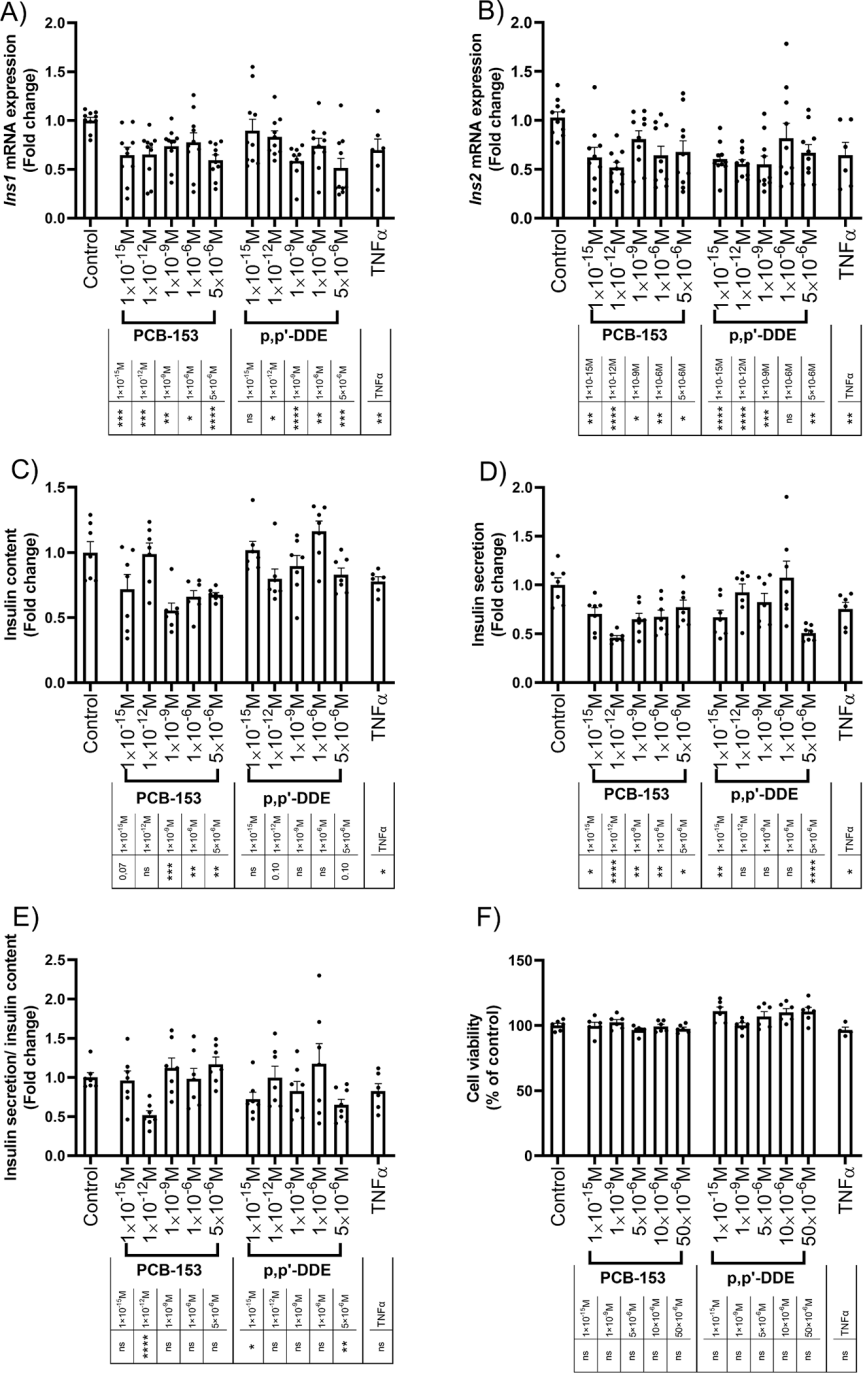
PCB-153 and p,p’-DDE reduce insulin production and secretion in β-cells. Pancreatic β-cells were treated with DMSO, POPs, or TNFα for 48 h as follows; after 45 h of culture with 11 mM glucose RPMI-1640 medium, cells were cultured in a low glucose (2 mM) medium for 1 h. Then, cells were cultured in a high glucose (20 mM) media for 2 h to stimulate their insulin production. At the end of this period, media were collected to assess insulin secretion, pancreatic β-cells were harvested to measure insulin content and mRNA expression of insulin genes. In another set of experiments, viability of pancreatic β-cells treated with DMSO, POPs or TNFα was assessed. In these cell viability experiments, two additional concentrations of POPs were included (10 × 10^−6^M and 50 × 10^−6^M). A-B: mRNA levels of *Ins1* (A) and *Ins2* (B) (n = 10 except for TNFα, n = 6). C: Intracellular insulin content (n = 7). D: Secreted insulin (n = 7). E: Ratio between secreted and intracellular insulin (n = 7). F: Cell viability (n = 6 except for TNFα, n = 4). In all figures, Control corresponds to DMSO-treated cells. TNFα (10 ng/mL) was used as a positive control. All data points represent biological replicates. Data are presented as the mean with SEM. Statistically significant difference vs. Control: *P < 0.05, **P < 0.01, ***P < 0.001 and ****P < 0.0001. ns means not significant.

To better understand the impact of POPs on β-cells, we investigated whether POPs could directly regulate the production of insulin in an experimental model using INS-1E cells, a widely used β-cell model in pancreatic research (Merglen et al. 2004). To determine whether POPs from different categories could trigger different effects on INS-1E cells, we selected one PCB and one organochlorine pesticide compound. We focused our investigations on PCB-153 and p,p’-DDE because both compounds had the highest OR for T1D/IS and were highly detectable in youths (Table 2). To mimic environmental doses of exposure, we treated cells with PCB-153 and p,p’-DDE at concentrations ranging from 1 × 10^−15^ M to 5 × 10^−6^ M. First, we explored whether PCB-153 and p,p’-DDE could affect the two insulin genes *Ins1* and *Ins2*, which are both involved in the production of insulin in INS-1E cells. Interestingly, both PCB-153 and p,p’-DDE robustly reduced the mRNA expression of *Ins1* and *Ins2* even at concentrations as low as 1 × 10^−15^ M (Fig. 1A and B). Next, we tested whether this decrease in insulin gene expression translated to a reduction in intracellular insulin level. For this, we assessed the insulin content of INS-1E cells using an insulin-specific ELISA kit. In line with the mRNA expression results, PCB-153 strongly reduced the content of insulin in INS-1E cells (Fig. 1C) whereas this effect was less pronounced following treatment with p,p’-DDE (Fig. 1C). Then, we investigated the ability of the pancreatic β-cells to secrete insulin in response to glucose. By assessing the insulin concentrations present in the culture media, we observed that the release of insulin was significantly reduced in cells exposed to PCB-153 at all tested concentrations (Fig. 1D) whereas p,p’-DDE had a marked effect only at the lowest and highest concentrations (Fig. 1D). When insulin secretion was assessed relative to intracellular insulin content (Fig. 1E), we found that PCB-153 treatment resulted in a constant ratio for most of the tested concentrations. This indicates that the observed decrease in intracellular insulin was accompanied by a similar decrease in insulin secretion in the cells. For p,p’-DDE treated cells, we observed a decreased ratio between secreted and intracellular insulin suggesting that the cells were less efficient at secreting the produced insulin compared to DMSO-treated cells (Fig. 1E). No effect on β-cell viability was observed with PCB-153 and p,p’-DDE treatment at the examined concentrations for 2 days (Fig. 1F).

### 3.3. Molecular mechanisms of POPs

**Fig. 2.**
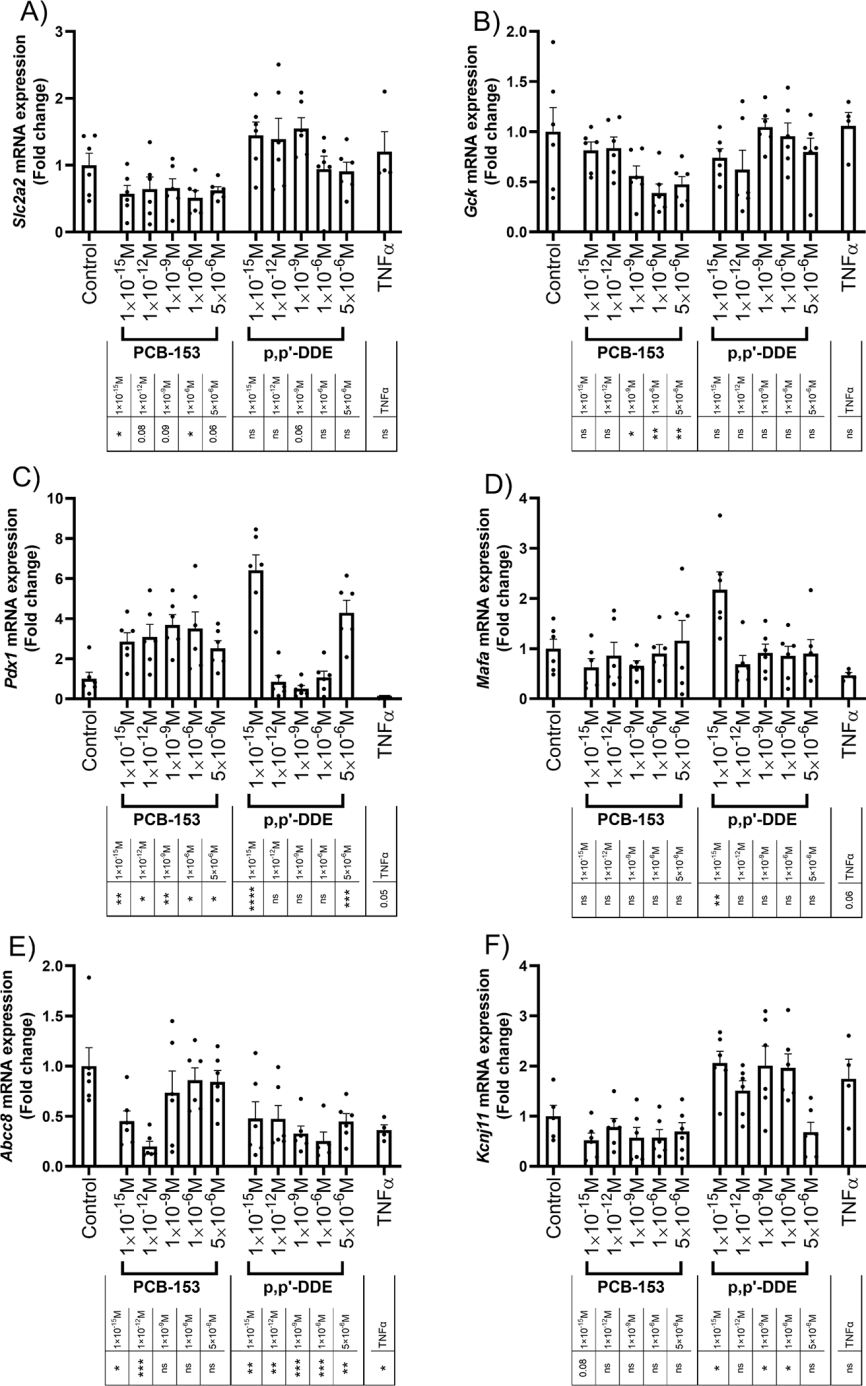
PCB-153 and p,p’-DDE alter mRNA expression of genes involved in glucose-stimulated insulin secretion. Pancreatic β-cells were treated with DMSO, POPs or TNFα for 48 h to determine mRNA expression, as described in Fig. 1. A-B: mRNA levels of the GLUT2 gene *Slc2a2* (A) and glucokinase gene *Gck* (B) (n = 6 except for TNFα, n = 4). C-D: mRNA levels of transcription factors *Pdx1* (C) and *Mafa* (D) (n = 6 except for TNFα, n = 4). E-F: mRNA levels of potassium channel genes *Abcc8* (E) and *Kcnj11* (F) (n = 6 except for TNFα, n = 4). In all figures, Control corresponds to DMSO-treated cells. TNFα (10 ng/mL) was used as a positive control. All data points represent biological replicates. Data are presented as the mean with SEM. Statistically significant difference vs. Control: *P < 0.05, **P < 0.01, ***P < 0.001 and ****P < 0.0001. ns means not significant.

To better understand the mechanistic modes of action of PCB-153 and p,p’-DDE, we assessed the mRNA expression of the glucose transporter GLUT2 (encoded by *Slc2a2*) and glucokinase (encoded by *Gck*), often referred to as “the glucose sensor”, with a critical role in the initiation of glucose-induced insulin secretion in pancreatic β-cells (Matschinsky and Wilson 2019). Interestingly, PCB-153, but not p,p’-DDE, reduced the expression level of *Slc2a2* (Fig. 2A) and *Gck* (Fig. 2B). Further, we studied the pancreatic duodenal homeobox 1 (PDX1) and V-maf musculoaponeurotic fibrosarcoma oncogene homolog A (MAFA), which are two transcription factors for insulin gene expression and pancreatic cell development (Zhu et al. 2017). PCB-153 significantly increased the expression level of *Pdx1* at all doses whereas similar effect was only observed with the lowest and highest dose of p,p’-DDE (Fig. 2C). No significant effect on *Mafa* expression was found, except for p,p’-DDE at the lowest dose (Fig. 2D). Finally, we observed no major impact of PCB-153 or p,p’-DDE on mRNA levels of *Pcsk1/3* or *Pcsk2*, two proteins converting proinsulin into insulin (Fig S2A and B).

Next, we assessed genes involved in the regulation of insulin secretion. We investigated the ATP-sensitive potassium channel regulating insulin secretion, which is composed of two distinct subunits, a regulatory subunit, the sulfonylurea receptor-1 (SUR1, encoded by *Abcc8*) and an inwardly rectifying ion channel forming the pore (KIR6.2, encoded by *Kcnj11*). Of note, p,p’-DDE treatment caused a significant reduction in *Abcc8* expression at all doses (Fig. 2E), which was associated with an up-regulation of *Kcnj11* (Fig. 2F). In contrast to p,p’-DDE, PCB-153 reduced the mRNA expression of *Abcc8* at the lowest doses only (Fig. 2E), without any increase of *Kcnj11* expression (Fig. 2F). We next asked whether PCB-153 and p,p’-DDE could impair voltage-gated calcium channels (CAV). To this end, we focused on CAV1.3 (encoded by *Cacna1d*) and CAV2.2 (encoded by *Cacna1b)* and found that neither PCB-153 nor p,p’-DDE affected their expression (Fig S2C and D). Finally, we investigated genes involved in exocytosis like *Stx1a*, *Snap25,* and *Sytl4* and found no significant impact of PCB-153 and p,p’-DDE (Fig S2E–G).

### 3.4. POPs and β-cell viability in vitro

**Fig. 3.**
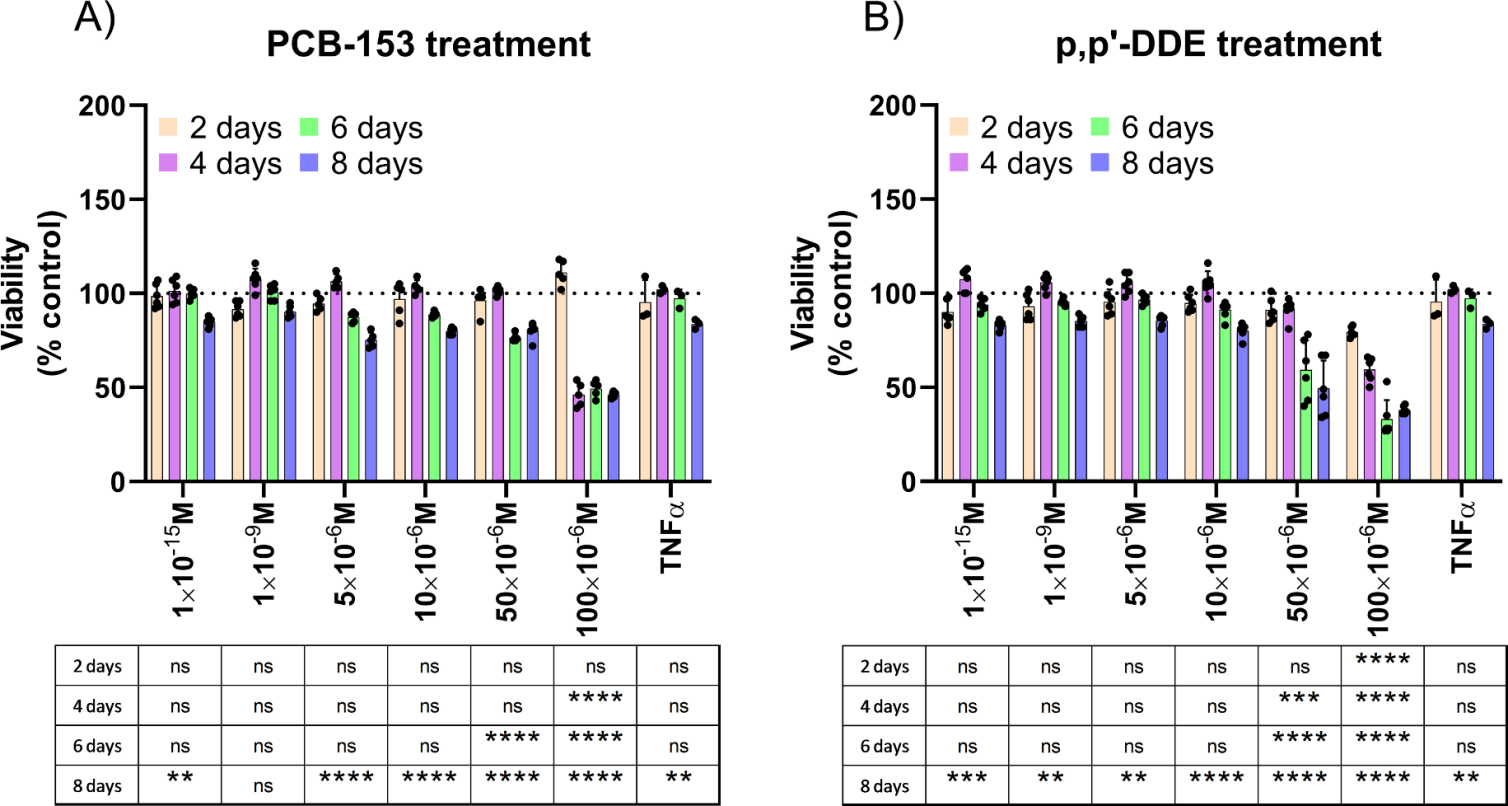
PCB-153 and p,p’-DDE progressively destroy pancreatic β-cells. Pancreatic β-cells were treated with DMSO, POPs,or TNFα for 8 days. Cells were plated and cultured in 11 mM glucose RPMI-1640 medium with DMSO, PCB-153, p,p’-DDE, or TNFα. Every two days, viability of the cells was assessed using AlamarBlue. A: Viability of PCB-153 treated cells (n = 6 except for TNFα, n = 4). B: Viability of p,p’-DDE treated cells (n = 6 except for TNFα, n = 4). In all figures, data are presented relative to the viability of DMSO treated cells (dashed line). TNFα (10 ng/mL) was used as a positive control. All data points represent biological replicates. Data are presented as the mean with SEM. Statistically significant difference vs. Control: *P < 0.05, **P < 0.01, ***P < 0.001 and ****P < 0.0001. ns means not significant.

The destruction of pancreatic β-cells is a critical feature of T1D, for which the etiology remains poorly known (Roep et al. 2021). We therefore asked whether a chronic treatment with PCB-153 or p,p’-DDE could affect the viability of β-cells. To this end, we performed a follow-up study of INS-1E cells and assessed their viability every 2 days. Confirming our previous data (Fig. 1F), treatment with PCB-153 and p,p’-DDE for 2 days did not affect cell viability at 1 × 10^−15^ M, 1 × 10^−9^ M, 5 × 10^−6^ M, and 50 × 10^−6^ M (Fig. 3A and B). However, when cells were exposed to a higher dose (100 × 10^−6^ M) of p,p’-DDE, cell viability was significantly reduced by about 20% (Fig. 3B). By day 6, PCB-153 and p, p’-DDE at 50 × 10^−6^ M and 100 × 10^−6^ M decreased the viability of β-cells by 25–60% (Fig. 3A and B). After 8 days of exposure, almost all PCB-153 and p,p’-DDE concentrations caused a significant destruction of β-cells (Fig. 3A and B).

## 4. Discussion

Using data and biological samples from SEARCH-CC, we explored for the first time the role of POPs in youth-onset T1D in a relatively large number of well-phenotyped individuals. In addition, we used an experimental model to delineate the impacts of POPs on β-cells. We found that increased plasma concentrations of p,p’-DDE, *trans*-nonachlor, and PCB-153 are associated with higher odds of having T1D/IS, but not T1D/IR. Among POPs with detection rates between 20 and 70%, only p,p’-DDT showed a similar association. Translating our human data to an experimental level, PCB-153 and p,p’-DDE significantly reduce intracellular insulin content and insulin secretion in INS-1E cells. Mechanistically, both PCB-153 and p,p’-DDE decrease the mRNA expression of *Ins1* and *Ins2.* In addition, while PCB-153 robustly impairs the expression of genes involved in glucose sensing (*Slc2a2* and *Gck*), the effect of p,p’-DDE mainly focuses on the ATP-sensitive potassium channel (*Abcc8* and *Kcnj11*). During long-term exposure with PCB-153 and p,p’-DDE, the viability of INS-1E cell decreases progressively.

There are very few studies on POPs and T1D in youth. Previously, women exposed to POPs during pregnancy did not have children with a higher risk of T1D (Rignell-Hydbom et al. 2010). In addition, in newborn infants with HLA genotypes conferring susceptibility to T1D, POP exposure was not associated with the development of T1D at 4 years of age (Salo et al. 2019). Interestingly, by stratifying T1D participants according to their insulin sensitivity, we found that POPs are associated with T1D/IS, but not T1D/IR. As compared to T1D/IS, youths with T1D/IR had a larger adipose tissue pool as indicated by a higher BMI and waist circumference. This increased adipose tissue pool gives T1D/IR individuals the capacity to store large amounts of POPs, thus facilitating the removal of POPs from the bloodstream. Indeed, POPs are lipophilic chemicals that mainly bioaccumulate in fatty-rich tissues, and weight gain (i.e. increased adipose tissue pool) is known to decrease circulating POP levels (Lim et al. 2011). This elimination of circulating POPs has also been reported during gestational weight gain (Vafeiadi et al. 2014). On the opposite, weight loss (i.e. reduced adipose tissue pool), either by diet (Imbeault et al. 2002) or bariatric surgery (Kim et al. 2011), increases the amount of POPs in the bloodstream. This increased ability of POP bioaccumulation and removal from the blood circulation, associated with increased body weight, may explain why youths with T1D/IR had lower serum POP levels than youths with T1D/IS, and why no association between circulating POPs and T1D/IR was found. In accordance, the detection values of many POPs, especially for PCBs, were higher in T1D/IS compared to T1D/IR. It is further likely that the increased POP storage in adipose tissue contributes to the development of insulin resistance (Ruzzin et al. 2010; Ibrahim et al. 2011; Lee et al. 2014). In our study, we were unable to assess the bioaccumulation of POPs in T1D/IS and T1D/IR because of the lack of adipose tissue biopsies. Nevertheless, our findings highlight the importance of performing in-depth phenotyping of T1D to better delineate the contribution of POPs to the disease. In this regard, future studies using human adipose tissue samples will bring important new knowledge.

**Fig. 4.**
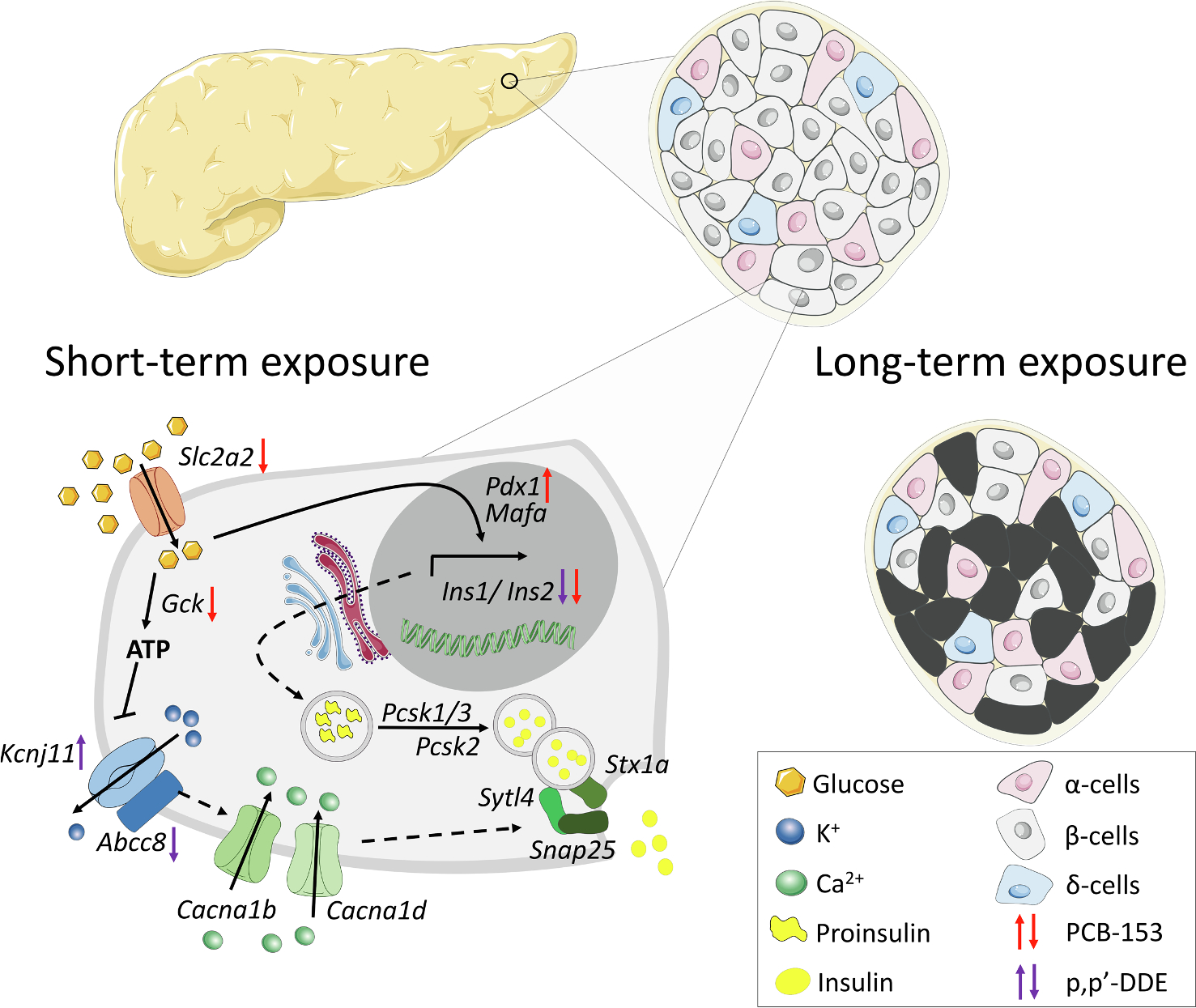
Impacts of POPs on pancreatic β-cells. Short-term exposure (2 days) to PCB-153 and p, p’-DDE impairs critical genes involved in the regulation of glucose-stimulated insulin production and secretion. While this POP exposure had no effect on cell viability, long-term exposure (over 2 days) to PCB-153 and p,p’-DDE progressively causes β-cell destruction (based on results presented in Fig. 3). Arrows for PCB-153 (red) and p,p’-DDE (purple) indicate whether the majority of tested concentrations affects the gene expression (based on results presented in Fig. 2 and S2). *Slc2a2* encodes GLUT2, *Gck* encodes glukokinase, *Kcnj11* encodes KIR6.2, *Abcc8* encodes SUR1, *Cacna1b* encodes CAV2.2, *Cacna1d* encodes CAV1.3, *Pdx1* encodes PDX1, *Mafa* encodes MAFA, *Pcsk1/3* encodes PC1/3, *Pcsk2* encodes PC2, *Sytl4* encodes granuphilin, *Stx1* encodes syntaxin and *Snap25* encodes SNAP25.

The origins and mechanisms of β-cell dysfunction in T1D remain unclear (Sherry et al. 2005). Previously, newly diagnosed patients with T1D were found to have ~ 50% lower insulin secretion in response to a meal as compared to nondiabetic control individuals (Steele et al. 2004). Interestingly, when islets isolated from T1D patients were incubated *in vitro*, the reduced glucose-stimulated insulin secretion was restored after 3–6 days of culture, thereby suggesting that the β-cells of T1D individuals are impaired by systemic factor(s) (Krogvold et al. 2015). In the present study, using an experimental model of glucose-stimulated insulin secretion for assessing β-cell dysfunction, we found that PCB-153 and p,p’-DDE treatment (1 × 10^−15^ M to 5 × 10^−6^ M) for 2 days strongly impair insulin production and secretion without affecting β-cell viability. Mechanistically, our experimental findings unveil that both PCB-153 and p,p’-DDE reduced the expression of *Ins1* and *Ins2*, the two genes encoding insulin. Similar down-regulation of mRNA expression of *Ins1* and *Ins2* transcripts was recently observed in INS-1E cells exposed to p,p’-DDE, and p,p’-DDT for one month (Pavlikova et al. 2019) and in pancreatic β-cells from mice exposed to Aroclor 1254, a mixture of PCBs (Xi et al. 2019). Of note, the decrease in *Ins1* and *Ins2* mRNA levels observed in our study was not paralleled by a reduction of the transcription factors *Pdx1* and *Mafa,* which may indicate that PCB-153 and p,p’-DDE regulate *Ins1* and *Ins2* genes through DNA methylation (Kuroda et al. 2009). Such epigenetic regulation caused by POPs has been described previously (Lind et al. 2013; van den Dungen et al. 2017), including a DNA methylation of the pancreatic *Ins2* promoter in mice exposed to Aroclor 1254 (Xi et al. 2019). Our *in vitro* investigations further reveal that PCB-153 and p,p’-DDE can have different molecular mechanisms of action on pancreatic β-cells. PCB-153 reduces the glucose-sensing capacity of the β-cells by decreasing *Slc2a2* and *Gck* expression. This impaired glucose sensing may contribute to the low insulin production and secretion found in β-cells treated with PCB-153. In contrast to PCB-153, p,p’-DDE does not affect genes involved in the transport (*Slc2a2*) and utilization of glucose (*Gck*) within β-cells. Rather, p,p’-DDE appears to mainly affect the ATP-sensitive potassium channel (*Abcc8* and *Kcnj11*), which supports our finding that the insulin secretion efficiency of cells treated with p,p’-DDE is impaired. Combined together, our experimental findings demonstrate that PCB-153 and p,p’-DDE can directly impair pancreatic β-cells by targeting similar (i.e. *Ins1* and *Ins2*) and different genes (Fig. 4). In a broader perspective, as humans are chronically exposed to a large number of PCBs and organochlorine pesticides, it is conceivable that these environmental chemicals cause a multifaceted attack on pancreatic β-cells leading, over time, to their failure.

The low dose and non-monotonicity of endocrine-disrupting chemicals represent a key issue in environmental toxicology (Vandenberg et al. 2012). Our findings show that the deleterious effects of PCB-153 and p,p’-DDE on pancreatic β-cells occur at very low doses of exposure. For instance, INS-1E cells treated with concentrations as low as 1 × 10^−15^ M exhibited strong impairments of insulin production and secretion. These data are in line with Lee et al. who reported, in the same pancreatic INS-1E cell line, a significant impairment of insulin content and secretion after exposure to organochlorine pesticides (p,p’-DDT, *trans*-nonachlor, β-hexachlorocyclohexane) and PCB mixture (Aroclor 1254) at 1 × 10^−12^ M and 1 × 10^−11^ M concentrations (Lee et al. 2017). As compared to INS-1E cells, 3T3-L1 adipocytes treated with PCBs and organochlorinated pesticides showed impaired insulin-stimulated glucose uptake at higher concentrations; 1 × 10^−6^ M and 1 × 10^−9^ M, respectively (Ruzzin et al. 2010). Altogether these data show that pancreatic β-cells are highly sensitive to POPs. Moreover, they indicate that the health risks associated with POP exposure are unlikely to be captured by a traditional risk assessment approach, which assumes that the dose–response curve of a chemical is monotonic.

Finally, since the environmental origins causing β-cell destruction remain poorly known, we further investigated whether long-term treatment (8 days) with POPs could decrease β-cell viability. Interestingly, our data showed that both PCB-153 and p,p’-DDE cause a progressive β-cell loss. After 8 days of POP treatment, INS-1E cell viability was significantly decreased, even at very low doses of exposure. Our findings support previous studies reporting increased β-cell apoptosis in mice treated with Aroclor 1254 (Fang et al. 2020) and are in line with other studies on POPs showing that TCDD, a dioxin compound, has a direct cytotoxic effect on INS-1E cells (Piaggi et al. 2007) and induces a progressive cell necrosis and apoptosis on isolated rat pancreatic islets (Novelli et al. 2021). Taken together, these findings strongly support a role of POPs in β-cell destruction.

### 4.1. Strengths and limitations

Major strengths of our study include the comprehensive phenotyping of a large number of youths with T1D, as well as the combination of human and *in vitro* studies, which allows a better determination of the causative role of POPs on T1D and the mechanisms involved. While we demonstrated the impacts of some PCBs and organochlorine pesticides on T1D, whether other PCBs and organochlorine pesticide compounds and POPs, like polybrominated diphenyl ethers (PBDEs) and perfluoroalkyl substances (PFAS), contribute to T1D in youths remain to be investigated. One potential limitation to our study is the fasting human samples, which may miss manifestation of dysglycemia in the participants (no provocative tests such as OGTT were performed). However, this issue is likely limited given the young age of the cohort.

## 5. Conclusion

In conclusion, our findings reveal a potential role of certain POPs, still circulating globally, in T1D in youth and provide *in vitro* evidence for a direct, and detrimental, effect of POPs on pancreatic β-cells.

## Supplementary Material

Supp.

## Figures and Tables

**Table 1:** Participant characteristics.

	Control	Type 1 diabetes Insulin Sensitive	Type 1 diabetes Insulin Resistant	p-values		
	(C)	(IS)	(IR)	C vs. IS	C vs. IR	IS vs. IR

	N = 112	N = 182	N = 148			
**SOCIO-ECONOMIC CHARACTERISTICS**						
**Age (years), mean ± sd**	14.2 ± 2.8	13.5 ± 2.7	16.1 ± 3.3	0.033	<0.0001	<0.0001
**Gender, n (%)**				0.04	0.70	0.086
**Female**	67 (60%)	86 (47%)	84 (57%)			
**Male**	45 (40%)	96 (53%)	64 (43%)			
**Race/Ethnicity, n (%)**				<0.0001	0.0016	0.16
**Non-Hispanic White**	61 (54%)	149 (82%)	109 (74%)			
**African American**	31 (28%)	17 (9%)	17 (11%)			
**Hispanic**	20 (18%)	16 (9%)	22 (15%)			
**Site, n (%)**				0.11	0.011	0.23
**Colorado**	65 (58%)	123 (68%)	109 (74%)			
**South Carolina**	47 (42%)	59 (32%)	39 (26%)			
**Parental Education, n (%)** ^a^				0.022	0.39	0.32
**Less than High School**	10 (9%)	8 (4%)	8 (5%)			
**High School graduate**	21 (19%)	18 (10%)	20 (14%)			
**Some College**	33 (30%)	51 (28%)	50 (34%)			
**Bachelor’s Degree or more**	47 (42%)	104 (58%)	70 (47%)			
**METABOLIC CHARACTERISTICS**						
**Duration of Type 1 diabetes (years), median (IQR)**	-	1.8 (0.5, 5.4)	3.6 (1.3, 6.7)	-	-	0.0006
**BMI z score, mean ± sd**	0.4 ± 0.9	0.2 ± 0.8	0.9 ± 0.8	0.046	<0.0001	<0.0001
**Waist Circumference (cm), mean ± sd**	72.9 ± 8.1	70.8 ± 7.5	85.5 ± 10.6	0.032	<0.0001	<0.0001
**Total Cholesterol (mg/dL), mean ± sd**	160.6 ± 25.2	163.4 ± 28.3	180.6 ± 41.7	0.39	<0.0001	<0.0001
**LDL (mg/dL), mean ± sd**	95.2 ± 24.2	95.2 ± 23.8	108.5 ± 32.6	0.99	0.0002	<0.0001
**HDL (mg/dL), mean ± sd**	50.6 ± 10.8	56.0 ± 11.2	52.4 ± 13.3	<0.0001	0.23	0.0076
**Triglycerides (mg/dL), median (IQR)**	69 (49; 96)	56 (44; 71)	82 (62; 105)	<0.0001	0.0023	<0.0001
**HbA1c (%), mean ± sd**	5.2 ± 0.3	7.6 ± 1.3	9.1 ± 2.1	<0.0001	<0.0001	<0.0001
**Fasting C-peptide (ng/ml), median (IQR)**	1.7 (1.2; 2.1)	0.3 (0.2; 0.9)	0.2 (0.2; 0.6)	<0.0001	<0.0001	0.015
**GADA** ^b^	-	22.5 (0.0, 145.0)	74.4 (3.5, 378.3)	-	-	0.0004
**IA-2A** ^b^	-	20.2 (0.0, 268.5)	34.0 (3.6, 339.4)	-	-	0.039
**ZnT8**	-	0.1 (0.0, 0.3)	0.1 (0.0, 0.3)	-	-	0.97
**Insulin sensitivity, mean ± sd**	12.2 ± 2.4	10.6 ± 1.7	6.2 ± 1.5	<0.0001	<0.0001	<0.0001
**Pubic Tanner stage, mean ± sd**	3.8 (1.3)	3.3 (1.4)	4.3 (1.0)	0.0006	0.0006	<0.0001

a For Parental Education both the control and T1D/IS groups have one participant that did not provide data.

b Controls who had an elevated value for GADA or IA-2A as well as deteriorated insulin sensitivity were excluded (n = 16 for GADA and IA-2A and n = 34 for insulin sensitivity). p-values are from *t*-test when means are presented, Kruskal-Wallis test when medians are presented, and Exact tests for categorical measures.

**Table 2:** Odds ratio of type 1 diabetes with normal insulin sensitivity or insulin resistance according to tertiles of lipid adjusted POP concentrations with detection rates over 70%.

		Type 1 diabetes			
Compounds	Detection Rate	Insulin sensitive N = 182		Insulin resistant N = 148	
		OR (95% CI)	P for trend	OR (95% CI)	P for trend

**p,p’-DDE**	99.6%		0.01		0.29
**2nd tertile**		2.0 (1.0, 3.8)		1.3 (0.6, 2.7)	
**3rd tertile**		2.4 (1.2, 5.0)		0.7 (0.3, 1.4)	
***trans*-Nonachlor**	78.3%		0.02		0.68
**2nd tertile**		2.5 (1.3, 5.0)		1.4 (0.7, 2.8)	
**3rd tertile**		2.3 (1.1, 5.1)		0.8 (0.4, 1.8)	
**Hexachlorobenzene**	100%		0.25		0.60
**2nd tertile**		0.8 (0.4, 1.6)		1.0 (0.5, 2.1)	
**3rd tertile**		0.7 (0.3, 1.3)		1.2 (0.6, 2.7)	
**SUM of OC** ^a^			0.31		0.38
**2nd tertile**		1.3 (0.7, 2.6)		1.0 (0.5, 2.0)	
**3rd tertile**		1.4 (0.7, 2.8)		0.7 (0.3, 1.5)	
**PCB-28**	82.6%		0.34		0.14
**2nd tertile**		0.8 (0.4, 1.6)		1.0 (0.5, 2.1)	
**3rd tertile**		0.7 (0.4, 1.4)		1.8 (0.8, 3.6)	
**PCB-153**	78.7%		0.02		0.79
**2nd tertile**		1.8 (0.9, 3.4)		1.1 (0.5, 2.2)	
**3rd tertile**		2.3 (1.1, 4.6)		1.1 (0.5, 2.3)	
**SUM PCB** ^a^			0.40		0.98
**2nd tertile**		1.0 (0.5, 1.9)		0.7 (0.3, 1.4)	
**3rd tertile**		1.4 (0.7, 2.7)		1.0 (0.5, 2.1)	
**SUM OC & PCB** ^a^			0.22		0.56
**2nd tertile**		1.2 (0.6, 2.3)		1.0 (0.5, 2.1)	
**3rd tertile**		1.6 (0.8, 3.1)		0.8 (0.4, 1.7)	

Odds ratios are from logistic regression models with the outcome being T1D with normal insulin sensitivity or T1D with insulin resistance as defined in Materials and Methods. The odds ratios use the control group and the lowest tertile of the POP concentration as reference groups. All models were adjusted for age at sampling, health insurance, parental education, race/ethnicity, sex, pubic Tanner stage and site as predictors. POP compounds use a lipid-adjusted measure for the compound and include total lipids as a covariate in the models. Models for each subtype are run separately and compared to Controls. P-value from trend is from a similar model with tertiles modelled as ordinal and tests for a linear trend across the tertiles. For Controls, N = 112. Twelve participants with T1D were excluded from the analyses due to missing autoantibodies or insulin sensitivity data.

a SUM of OC is the sum of p,p’-DDE, *trans*-Nonachlor, and Hexachlorobenzene. SUM of PCB is the sum of PCB-28 and PCB-153. OC means organochlorine pesticides.
