# Supplementary material for: Associations between persistent organic pollutants and type 1 diabetes in youth
Source: Environ Int. Author manuscript; Available in PMC 2025 Jan 3. (PMC11696922; doi:10.1016/j.envint.2022.107175)
Supplement: Supp. [file NIHMS2040441-supplement-Supp_.docx]

**Table S1.** Overview of analyzed POPs

| **Analytes** | **Abbreviation** |
| --- | --- |
| ***Organochlorine pesticides*** | |
| α-Hexachlorocyclohexane | α-HCH |
| β-Hexachlorocyclohexane | β-HCH |
| γ-Hexachlorocyclohexane | γ-HCH |
| Heptachlor |  |
| cis-Heptachlor epoxide |  |
| Oxychlordane | oxy-CD |
| cis-Chlordane | cis-CD |
| trans-Chlordane | trans-CD |
| cis-Nonachlor | cis-NC |
| trans-Nonachlor | trans-NC |
| 2,4'-Dichlorodiphenyldichloroethane | o,p'-DDD |
| 4,4'-Dichlorodiphenyldichloroethane | p,p'-DDD |
| 2,4'-Dichlorodiphenyldichloroethylene | o,p'-DDE |
| 4,4'-Dichlorodiphenyldichloroethylene | p,p'-DDE |
| 2,4'-Dichlorodiphenyltrichloroethane | o,p'-DDT |
| 4,4'-Dichlorodiphenyltrichloroethane | p,p'-DDT |
| Hexachlorobenzene | HCB |
| ***Polychlorinated biphenyls*** | |
| 2,4,4'-Trichlorobiphenyl | PCB-28 |
| 2,2',5,5'-Tetrachlorobiphenyl | PCB-52 |
| 2,4,4',5-Tetrachlorobiphenyl | PCB-74 |
| 2,2',4,4',5-Pentachlorobiphenyl | PCB-99 |
| 2,2',4,5,5'-Pentachlorobiphenyl | PCB-101 |
| 2,3,3',4,4'-Pentachlorobiphenyl | PCB-105 |
| 2,3',4,4',5-Pentachlorobiphenyl | PCB-118 |
| 2,2',3,3',4,4'-Hexachlorobiphenyl | PCB-128 |
| 2,2',3,4,4',5'-Hexachlorobiphenyl | PCB-138 |
| 2,2',3,4',5',6-Hexachlorobiphenyl | PCB-149 |
| 2,2',4,4',5,5'-Hexachlorobiphenyl | PCB-153 |
| 2,3,3',4,4',5-Hexachlorobiphenyl | PCB-156 |
| 2,3,3',4,4',5'-Hexachlorobiphenyl | PCB-157 |
| 2,3',4,4',5,5'-Hexachlorobiphenyl | PCB-167 |
| 3,3',4,4',5,5'-Hexachlorobiphenyl | PCB-169 |
| 2,2',3,3',4,4',5-Heptachlorobiphenyl | PCB-170 |
| 2,2',3,4,4',5,5'-Heptachlorobiphenyl | PCB-180 |
| 2,2',3,4,4',5',6-Heptachlorobiphenyl | PCB-183 |
| 2,2',3,4',5,5',6-Heptachlorobiphenyl | PCB-187 |
| 2,3,3',4,4',5,5'-Heptachlorobiphenyl | PCB-189 |
| 2,2',3,3',4,4',5,5'-Octachlorobiphenyl | PCB-194 |

**Table S2.** List of used primers

| **Gene** | **Forward** | **Reverse** |
| --- | --- | --- |
| ***Ins1*** | GGGGAACGTGGTTTCTTCTAC | CCAGTTGGTAGAGGGAGCAG |
| ***Ins2*** | CAGCACCTTTGTGGTTCTCA | CACCTCCAGTGCCAAGGT |
| ***Tbp*** | TGGTGTGCACAGGAGCCAAG | TTCACATCACAGCTCCCCAC |
| ***Pdx1*** | TGCTAATCCCCCTGCGTGCCTGTA | CTCCTCCGGTTCTGCGTATGC |
| ***Slc2a2*** | TGGGTTCCTTCCAGTTCG | AGGCGTCTGGTGTCGTATG |
| ***Gck*** | TGACAGAGCCAGGATGGAG | TCTTCACGCTCCACTGCC |
| ***Kcnj11*** | CCATGTCCTTCCTGTGCAGCTG | CCGCAACTCAGGACAAGGAATC |
| ***Abcc8*** | TTGCTGAAACTGTGGAAGGACTCAC | TTCAGGACCATCACTAGGTCTGCAC |
| ***Snap25*** | CAACGTGCAACAAAGATGCT | CAATGGGGGTGACTGACTCT |
| ***Stx1a*** | GCCCTCAGTGAGATCGAGAC | TACTTGACGGCCTTCTTGGT |
| ***Sytl4*** | GTCTGCTGAAGGAGGGACTG | GAGAATGCCGAGTTCTGGAG |
| ***Pcsk1/3*** | AGGCACCTCAGCTTCTGCAC | TCCACCAGAGCTTTGGCATT |
| ***Pcsk2*** | CCAAGTTGCAGCAGAACACG | TGCTGCAGGGCCATCTTTAT |
| ***Mafa*** | AGGAGGAGGTCATCCGACTG | CTTCTCGCTCTCCAGAATGTG |
| ***Cacna1d*** | TGGATTACGCACCTTGCAT | GCCGTCAGATCCCCAGAGA |
| ***Cacna1b*** | AAGGCGCTGCCCTACGT | GCCGATGATGGCGTAGATG |

**Table S3.** Limit of detection, detection rate and distribution of concentrations of persistent organic pollutants.

|  | | **Detection rate (%)** | | | | **Distribution of observed values** | | | | | |
| --- | --- | --- | --- | --- | --- | --- | --- | --- | --- | --- | --- |
| **Compound** | **Limit of detection (LOD)** | **All** | **Control** | **Insulin Sensitive** | **Insulin Resistant** | **10th percentile** | **25th percentile** | **50th percentile** | **75th percentile** | **90th percentile** | **max** |
| **o,p’-DDE** | 0.25 (0.22) | 40.5 | 34.82 | 37.91 | 47.97 | 0.27 | 0.4 | 0.53 | 0.76 | 1.02 | 10.14 |
| **p,p’-DDE** | 2.85 (9.02) | 99.55 | 99.11 | 99.45 | 100 | 135.4 | 195.2 | 285.59 | 416.93 | 609.5 | 6711.62 |
| **o,p’-DDD** | 1.15 (0.58) | 2.71 | 2.68 | 2.75 | 2.7 | 1.3 | 1.36 | 1.86 | 2.71 | 4.83 | 8.31 |
| **p,p’-DDD** | 3.13 (2.66) | 3.39 | 3.57 | 4.4 | 2.03 | 2.66 | 3.79 | 5.08 | 16.58 | 17.2 | 26.92 |
| **o,p’-DDT** | 1.36 (1.22) | 9.05 | 5.36 | 9.34 | 11.49 | 1.86 | 2.71 | 4.55 | 5.95 | 8.55 | 23.99 |
| **p,p’-DDT** | 2.44 (2.13) | 37.33 | 28.57 | 40.66 | 39.86 | 4.92 | 5.94 | 7.82 | 11.01 | 19.85 | 243.39 |
| **α-HCH** | 3.56 (2.27) | 3.62 | 3.57 | 2.75 | 4.73 | 6.47 | 7.18 | 8.93 | 13.44 | 18.7 | 19.06 |
| **γ-HCH** | 28.10 (0.00) | 22.62 | 31.25 | 17.58 | 22.3 | 29.36 | 30.47 | 35.53 | 40.61 | 53.6 | 67.2 |
| **β-HCH** | 4.56 (2.93) | 39.37 | 33.93 | 45.05 | 36.49 | 6.52 | 8.89 | 11.94 | 18.64 | 29.01 | 102.96 |
| **Heptachlor** | 0.83 (1.46) | 50.45 | 50.89 | 50.55 | 50 | 0.96 | 1.6 | 2.94 | 4.68 | 6.53 | 51.25 |
| **trans-Chlordane** | 0.83 (1.05) | 38.24 | 41.96 | 36.81 | 37.16 | 0.82 | 1.52 | 2.72 | 4.87 | 8.19 | 48.91 |
| **cis-Chlordane** | 0.87 (1.00) | 23.53 | 23.21 | 25.82 | 20.95 | 0.83 | 1.21 | 2.01 | 3.44 | 5.2 | 28.65 |
| **trans-Nonachlor** | 2.05 (2.61) | 78.28 | 75 | 84.62 | 72.97 | 4.82 | 7.55 | 13.23 | 25.49 | 56.35 | 290.57 |
| **cis-Nanochlor** | 1.01 (1.11) | 19.46 | 23.21 | 17.03 | 19.59 | 1.4 | 1.78 | 3.16 | 5.62 | 9.91 | 31.41 |
| **Oxychlordane** | 7.67 (7.55) | 18.33 | 16.96 | 22.53 | 14.19 | 13.03 | 19.88 | 34.28 | 59.89 | 98.32 | 227.13 |
| **cis-Heptachlor epoxide** | 2.09 (2.05) | 43.44 | 34.82 | 46.7 | 45.95 | 3.17 | 5.04 | 7.6 | 13.83 | 33.88 | 202.66 |
| **Hexachlorobenzene** | 2.11 (3.01) | 100 | 100 | 100 | 100 | 120.82 | 140.71 | 170.78 | 212.92 | 279.55 | 629.66 |
|  |  |  |  |  |  |  |  |  |  |  |  |
| **PCB-28** | 1.58 (0.99) | 82.58 | 78.57 | 83.52 | 84.46 | 12.75 | 18.06 | 25.38 | 32.8 | 41.16 | 149.98 |
| **PCB-52** | 9.78 (7.65) | 69.91 | 72.32 | 74.18 | 62.84 | 7.59 | 12.63 | 20.17 | 29.9 | 45.23 | 68.42 |
| **PCB-74** | 9.12 (7.09) | 37.33 | 42.86 | 42.31 | 27.03 | 7.28 | 8.63 | 11.95 | 18.15 | 30.17 | 94.24 |
| **PCB-99** | 3.71 (2.48) | 57.92 | 54.46 | 63.74 | 53.38 | 5.26 | 6.97 | 10.48 | 14.03 | 20.86 | 104.36 |
| **PCB-101** | 3.67 (2.44) | 50.23 | 49.11 | 52.75 | 47.97 | 6.74 | 8.25 | 10.86 | 14.9 | 20.85 | 139.3 |
| **PCB-105** | 5.13 (3.27) | 11.76 | 15.18 | 12.09 | 8.78 | 3.53 | 4.75 | 6.56 | 12.17 | 21.78 | 63.86 |
| **PCB-118** | 4.59 (2.98) | 60.86 | 58.04 | 65.38 | 57.43 | 9.33 | 12.34 | 16.3 | 23.33 | 35.37 | 222.4 |
| **PCB-128** | 7.44 (4.78) | 0.23 | 0 | 0 | 0.68 | 12.18 | 12.18 | 12.18 | 12.18 | 12.18 | 12.18 |
| **PCB-138** | 5.03 (2.73) | 56.79 | 54.46 | 60.44 | 54.05 | 12.06 | 15.87 | 23.14 | 34.23 | 48.89 | 166.53 |
| **PCB-149** | 6.25 (3.70) | 21.72 | 27.68 | 21.98 | 16.89 | 4.79 | 5.55 | 7.21 | 9.4 | 19.78 | 111.42 |
| **PCB-153** | 5.99 (3.38) | 78.73 | 75 | 83.52 | 75.68 | 15.87 | 21.52 | 30.21 | 45.99 | 64.71 | 177.5 |
| **PCB-156** | 9.43 (5.45) | 3.17 | 4.46 | 3.85 | 1.35 | 4.26 | 5.27 | 7.65 | 9.25 | 16.01 | 18.81 |
| **PCB-157** | 8.38 (5.51) | 0.45 | 0.89 | 0.55 | 0 | 4.44 | 4.44 | 4.82 | 5.19 | 5.19 | 5.19 |
| **PCB-167** | 9.86 (5.59) | 0.45 | 0 | 0.55 | 0.68 | 4.99 | 4.99 | 6.42 | 7.85 | 7.85 | 7.85 |
| **PCB-169** | 12.15 (6.58) | 0.23 | 0.89 | 0 | 0 | 3.89 | 3.89 | 3.89 | 3.89 | 3.89 | 3.89 |
| **PCB-170** | 10.17 (7.00) | 9.28 | 8.93 | 10.99 | 7.43 | 4.5 | 6.6 | 10.74 | 15.12 | 23.61 | 45.81 |
| **PCB-180** | 12.27 (10.30) | 35.07 | 36.61 | 40.11 | 27.7 | 9.56 | 16.66 | 28.31 | 42.87 | 70.12 | 141.87 |
| **PCB-183** | 14.44 (14.80) | 2.71 | 1.79 | 4.4 | 1.35 | 4.33 | 4.39 | 4.88 | 9.56 | 13.26 | 14.39 |
| **PCB-187** | 15.52 (13.76) | 9.73 | 12.5 | 11.54 | 5.41 | 4.82 | 6.02 | 10.52 | 16.59 | 30.64 | 77.89 |
| **PCB-189** | 8.85 (4.77) | 0.68 | 0 | 1.1 | 0.68 | 21.34 | 21.34 | 23.32 | 24.06 | 24.06 | 24.06 |
| **PCB-194** | 8.16 (6.30) | 9.28 | 8.93 | 12.64 | 5.41 | 4.54 | 6.5 | 11.29 | 17.83 | 24.54 | 39.87 |

LOD is expressed as mean (SD).

**Table S4.** Odds ratio of type 1 diabetes with normal insulin sensitivity or insulin resistance according to tertiles of lipid adjusted POP concentrations with detection rates between 20% and 70%.

|  |  | **Type 1 diabetes** | | | | |
| --- | --- | --- | --- | --- | --- | --- |
| **Compounds** | **Detection Rate** | **Insulin sensitive**  **N=182** | | | **Insulin resistant**  **N=148** | |
|  |  | **OR (95% CI)** | **P for trend** | **OR (95% CI)** | | **P for trend** |
| **o,p’-DDE** | 40.5% |  | 0.52 |  | | 0.54 |
| **2nd tertile** |  | 0.8 (0.4, 1.7) |  | 0.8 (0.3, 1.7) | |  |
| **3rd tertile** |  | 0.8 (0.4, 1.6) |  | 0.8 (0.4, 1.7) | |  |
| **p,p’-DDT** | 37.3% |  | 0.03 |  | | 0.13 |
| **2nd tertile** |  | 1.1 (0.5, 2.2) |  | 0.9 (0.4, 1.7) | |  |
| **3rd tertile** |  | 2.1 (1.0, 4.3) |  | 1.8 (0.9, 3.6) | |  |
| **γ-HCH** | 22.6% |  | 0.11 |  | | 0.57 |
| **2nd tertile** |  | 0.6 (0.2, 1.6) |  | 0.6 (0.2, 2.1) | |  |
| **3rd tertile** |  | 0.4 (0.2, 1.2) |  | 0.7 (0.2, 2.6) | |  |
| **β-HCH** | 39.4% |  | 0.59 |  | | 0.73 |
| **2nd tertile** |  | 0.8 (0.4, 1.6) |  | 0.8 (0.4, 1.9) | |  |
| **3rd tertile** |  | 1.3 (0.7, 2.7) |  | 1.2 (0.5, 2.6) | |  |
| **Heptachlor** | 50.5% |  | 0.98 |  | | 0.28 |
| **2nd tertile** |  | 1.2 (0.6, 2.5) |  | 2.1 (1.0, 4.7) | |  |
| **3rd tertile** |  | 0.9 (0.5, 1.9) |  | 1.5 (0.7, 3.1) | |  |
| **trans-Chlordane** | 23.5% |  | 0.68 |  | | 0.87 |
| **2nd tertile** |  | 0.8 (0.4, 1.7) |  | 1.4 (0.7, 2.9) | |  |
| **3rd tertile** |  | 0.8 (0.4, 1.6) |  | 1.1 (0.5, 2.4) | |  |
| **cis-Chlordane** | 19.5% |  | 0.93 |  | | 0.99 |
| **2nd tertile** |  | 1.0 (0.4, 2.2) |  | 1.0 (0.4, 2.4) | |  |
| **3rd tertile** |  | 1.2 (0.6, 2.5) |  | 1.0 (0.4, 2.3) | |  |
| **cis-Heptachlor epoxide** | 43.4% |  | 0.22 |  | | 0.64 |
| **2nd tertile** |  | 1.5 (0.7, 3.4) |  | 0.9 (0.4, 2.2) | |  |
| **3rd tertile** |  | 1.9 (0.9, 4.1) |  | 1.2 (0.5, 2.8) | |  |
| **PCB-52** | 69.9% |  | 0.30 |  | | 0.06 |
| **2nd tertile** |  | 1.4 (0.7, 3.0) |  | 0.4 (0.1, 1.3) | |  |
| **3rd tertile** |  | 0.9 (0.4, 1.7) |  | 0.4 (0.2, 1.0) | |  |
| **PCB-74** | 37.3% |  | 0.41 |  | | 0.07 |
| **2nd tertile** |  | 0.7 (0.3, 1.6) |  | 0.4 (0.1, 0.9) | |  |
| **3rd tertile** |  | 0.7 (0.3, 1.7) |  | 0.5 (0.2, 1.0) | |  |
| **PCB-99** | 57.9% |  | 0.83 |  | | 0.43 |
| **2nd tertile** |  | 0.7 (0.4, 1.5) |  | 0.7 (0.3, 1.5) | |  |
| **3rd tertile** |  | 1.5 (0.7, 3.1) |  | 0.7 (0.3, 1.6) | |  |
| **PCB-101** | 50.2% |  | 0.27 |  | | 0.73 |
| **2nd tertile** |  | 0.7 (0.3, 1.3) |  | 0.5 (0.2, 1.1) | |  |
| **3rd tertile** |  | 1.0 (0.5, 1.9) |  | 0.8 (0.4, 1.9) | |  |
| **PCB-118** | 60.9% |  | 0.17 |  | | 0.76 |
| **2nd tertile** |  | 1.4 (0.7, 2.8) |  | 1.1 (0.4, 2.7) | |  |
| **3rd tertile** |  | 1.5 (0.8, 3.0) |  | 0.9 (0.4, 2.0) | |  |
| **PCB-138** | 56.8% |  | 0.44 |  | | 0.24 |
| **2nd tertile** |  | 1.1 (0.6, 2.3) |  | 0.8 (0.3, 1.9) | |  |
| **3rd tertile** |  | 1.5 (0.8, 3.0) |  | 0.6 (0.3, 1.4) | |  |
| **PCB-149** | 21.7% |  | 0.50 |  | | 0.44 |
| **2nd tertile** |  | 0.8 (0.3, 1.8) |  | 0.7 (0.3, 1.7) | |  |
| **3rd tertile** |  | 1.4 (0.6, 3.0) |  | 0.7 (0.3, 1.6) | |  |

Odds ratios are from logistic regression models with the outcome being T1D with normal insulin sensitivity or T1D with insulin resistance as defined in Methods. The odds ratios use the control group and the lowest tertile of the POP concentration as reference groups. All models were adjusted for age at sampling, health insurance, parental education, race/ethnicity, sex, pubic Tanner stage and site as predictors. POP compounds use a lipid-adjusted measure for the compound and include total lipids as a covariate in the models. Models for each subtype are run separately and compared to Controls. P-value from trend is from a similar model with tertiles modelled as ordinal and tests for a linear trend across the tertiles. For Controls, N=112. Twelve participants with T1D were excluded from the analyses due to missing autoantibodies or insulin sensitivity data.

**Table S5.** Odds ratio of type 1 diabetes with normal insulin sensitivity or insulin resistance according to tertiles of non-lipid adjusted POP concentrations with detection rates over 70%.

|  |  | **Type 1 diabetes** | | | | |
| --- | --- | --- | --- | --- | --- | --- |
| **Compounds** | **Detection Rate** | **Insulin sensitive**  **N=182** | | | **Insulin resistant**  **N=148** | |
|  |  | **OR (95% CI)** | **P for trend** | **OR (95% CI)** | | **P for trend** |
| **p,p’-DDE** | 99.6% |  | 0.01 |  | | 0.68 |
| **2nd tertile** |  | 1.9 (1.0, 3.7) |  | 1.2 (0.6, 2.5) | |  |
| **3rd tertile** |  | 2.5 (1.2, 5.1) |  | 0.8 (0.4, 1.8) | |  |
| **trans-Nonachlor** | 78.3% |  | 0.01 |  | | 0.93 |
| **2nd tertile** |  | 2.3 (1.2, 4.5) |  | 1.5 (0.8, 3.1) | |  |
| **3rd tertile** |  | 2.7 (1.2, 6.0) |  | 0.9 (0.4, 2.1) | |  |
| **Hexachlorobenzene** | 100% |  | 0.23 |  | | 0.95 |
| **2nd tertile** |  | 1.5 (0.8, 3.0) |  | 1.0 (0.5, 2.1) | |  |
| **3rd tertile** |  | 0.7 (0.4, 1.3) |  | 1.0 (0.5, 2.0) | |  |
| **SUM of OC^a^** |  |  | 0.07 |  | | 0.30 |
| **2nd tertile** |  | 1.4 (0.7, 2.6) |  | 0.9 (0.5, 1.9) | |  |
| **3rd tertile** |  | 1.9 (0.9, 3.7) |  | 0.7 (0.3, 1.4) | |  |
| **PCB-28** | 82.6% |  | 0.32 |  | | 0.10 |
| **2nd tertile** |  | 0.7 (0.4, 1.3) |  | 0.9 (0.5, 1.9) | |  |
| **3rd tertile** |  | 0.7 (0.4, 1.4) |  | 1.8 (0.9, 3.8) | |  |
| **PCB-153** | 78.7% |  | 0.01 |  | | 0.98 |
| **2nd tertile** |  | 1.7 (0.9, 3.4) |  | 1.3 (0.7, 2.6) | |  |
| **3rd tertile** |  | 2.5 (1.2, 4.9) |  | 1.0 (0.5, 2.0) | |  |
| **SUM PCB^a^** |  |  | 0.10 |  | | 0.89 |
| **2nd tertile** |  | 1.6 (0.8, 3.2) |  | 1.0 (0.5, 2.0) | |  |
| **3rd tertile** |  | 1.8 (0.9, 3.5) |  | 1.1 (0.5, 2.2) | |  |
| **SUM OC & PCB^a^** |  |  | 0.12 |  | | 0.33 |
| **2nd tertile** |  | 1.5 (0.8, 3.0) |  | 0.8 (0.4, 1.7) | |  |
| **3rd tertile** |  | 1.7 (0.9, 3.3) |  | 0.7 (0.3, 1.4) | |  |

Odds ratios are from logistic regression models with the outcome being T1D with normal insulin sensitivity or T1D with insulin resistance as defined in Methods. The odds ratios use the control group and the lowest tertile of the POP concentration as reference groups. All models were adjusted for age at sampling, health insurance, parental education, race/ethnicity, sex, pubic Tanner stage and site as predictors. POP compounds use a non-lipid-adjusted measure for the compound and include total lipids as a covariate in the models. Models for each subtype are run separately and compared to Controls. P-value from trend is from a similar model with tertiles modelled as ordinal and tests for a linear trend across the tertiles. For Controls, N=112. Twelve participants with T1D were excluded from the analyses due to missing autoantibodies or insulin sensitivity data. ^a^SUM of OC is the sum of p,p’-DDE, trans-Nonachlor, and Hexachlorobenzene. SUM of PCB is the sum of PCB-28 and PCB-153. OC means organochlorine pesticides.

**Figure S1**


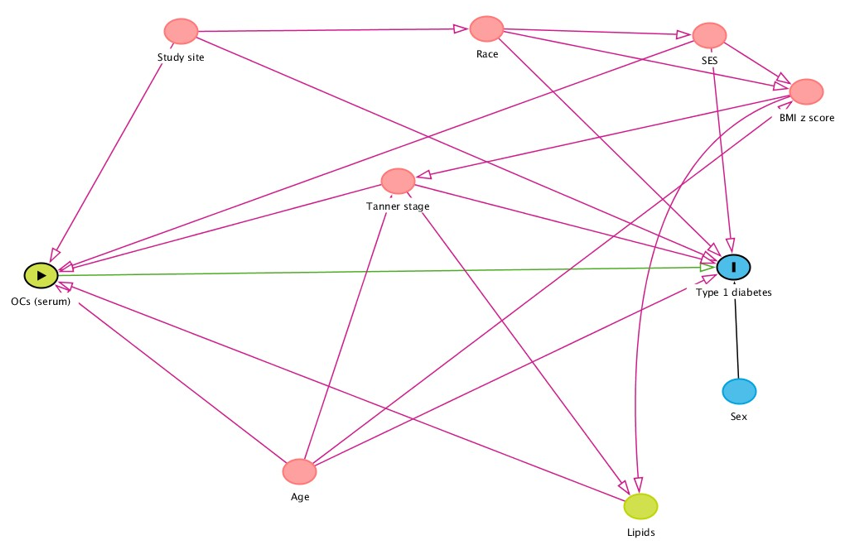


**Figure S1. The directed acyclic graph (DAG).**

We used a DAG informed approach for selection of a minimal sufficient set of factors for adjustment, blocking non-causal paths and leaving causal paths open. Multiple sufficient sets were identified, and we selected the set of factors for which the magnitude of the associations were likely higher (based on literature research) and where measurement of the factors was least likely to be influenced by misclassification. Our final adjustment set included: age at sampling, health insurance, parental education, race/ethnicity, sex, pubic Tanner stage, total lipids, and site. The DAG was constructed using the software program DAGitty. OC, organochlorine pesticides; SES, socioeconomic status. DAG developed for the PCBs was similar to OCs.

**Figure S2**

**Figure S2. PCB-153 and p,p’-DDE do not affect the mRNA level of genes involved in prohormone convertases, voltage-gated calcium channels or in exocytosis.**

Pancreatic β-cells were treated with DMSO, POPs or TNFα for 48 hours to measure mRNA expression as described in Figure 1.

A-B: mRNA levels of prohormone convertase PC1/3 gene *Pcsk1/3* (A) and PC2 gene *Pcsk2* (B) (n=6 except for TNFα, n=4).

C-D: mRNA levels of voltage-gated calcium channel CAV1.3 gene *Cacna1d* (C) and CAV2.2 gene *Cacna1b* (D) (n=6 except for TNFα, n=4).

E-G: mRNA levels of genes involved in exocytosis; *Stx1a* (E), *Snap25* (F) and *Sytl4* (G) (n=6 except for TNFα, n=4).

In all figures, Control corresponds to DMSO-treated cells. TNFα (10 ng/mL) was used as a positive control. All data points represent biological replicates. Data are presented as the mean with SEM. Statistically significant difference vs. Control: *P<0.05, **P<0.01, ***P<0.001 and ****P<0.0001. ns means not significant.
